# Prevalence of multimorbid degenerative lumbar spinal stenosis with knee and/or hip osteoarthritis: protocol for a systematic review and meta-analysis

**DOI:** 10.1186/s13643-020-01478-4

**Published:** 2020-10-07

**Authors:** James J. Young, Jan Hartvigsen, Rikke K. Jensen, Ewa M. Roos, Carlo Ammendolia, Carsten Bogh Juhl

**Affiliations:** 1 Center for Muscle and Joint Health, University of Southern Denmark, Campusvej 55, 5230 Odense M, Denmark; 2 Research Division, Canadian Memorial Chiropractic College, 6100 Leslie Street, Toronto, Canada; 3 Nordic Institute of Chiropractic and Clinical Biomechanics, Odense M, Denmark; 4 Rebecca MacDonald Centre for Arthritis and Autoimmune Diseases, Mount Sinai Hospital, Toronto, Canada; 5 Institute for Health Policy, Management and Evaluation, University of Toronto, Toronto, Canada; 6 Department of Physiotherapy and Occupational Therapy, Copenhagen University Hospital, and Gentofte, Herlev, Denmark

**Keywords:** Lumbar spinal stenosis, Hip osteoarthritis, Knee osteoarthritis, Multimorbidity, Prevalence, Systematic review protocol

## Abstract

**Background:**

Lumbar spinal stenosis (LSS) and knee and hip osteoarthritis (OA) are prevalent conditions in the aging population and published literature suggests they share many symptoms and often are present at the same time in patients. However, no prevalence estimates of multimorbid LSS and knee and/or hip OA are currently available. The primary objective of this systematic review is therefore to estimate the prevalence of multimorbid LSS with knee and/or hip OA using radiological, clinical, and combined case definitions.

**Methods:**

This systematic review protocol has been designed according to the guidelines from the Cochrane Collaboration and is reported according to the Preferred Reporting Items for Systematic Reviews and Meta-Analysis Protocols. A comprehensive search will be performed in the following databases: MEDLINE, EMBASE, CENTRAL, and CINAHL. Forward citation tracking will be performed in Web of Science. No restriction for publication date and language will be applied in the literature search, but only articles in English will be included. The search strategy will include the following domains: LSS, knee OA, and hip OA. Retrieved citations will be screened by two authors independently. Disagreements will be discussed until consensus, and a third reviewer will be consulted if consensus cannot be reached. Data extraction and assessment of risk of bias assessment will be done by two authors independently, using a standardized data extraction form and a modified risk of bias tool for prevalence studies. Meta-analysis estimating prevalence with 95% CI will be performed using a random effects model. Meta-regression analyses will be performed to investigate the impact of the following covariates: LSS clinical presentations, sample population, healthcare setting, risk of bias, and other patient characteristics on prevalence estimates for multimorbid LSS and knee and/or hip OA.

**Discussion:**

The results of this review will provide the first estimates of the prevalence of multimorbid LSS and hip and knee OA based on various case definitions. The impact of covariates such as LSS clinical presentations, sample population, healthcare setting, risk of bias, and patient characteristics on prevalence estimates will also be presented.

**Systematic review registration:**

PROSPERO, awaiting registration

## Background

Musculoskeletal health is considered an important prerequisite for healthy aging [1]. Unfortunately, musculoskeletal pain is a leading and growing cause of disability [2, 3]. Low back pain and osteoarthritis (OA) are among the most disabling chronic conditions globally, ranking as the first and twelfth causes of years lived with disability, respectively [4]. Chronic conditions are also the leading cause of the increased prevalence of multimorbidity (presence of two or more co-occurring diseases) among older individuals (estimated 67% of Americans over the age of 65) and individuals experience increased functional limitations with each additional chronic disease [5]. However, the impact of multimorbid musculoskeletal conditions on both patients and healthcare systems has not been extensively studied.

Lumbar spinal stenosis (LSS) is a lumbar spine condition that occurs with increasing age and is associated with substantial pain and disability in older adults [6]. LSS is considered one of the most burdensome spinal conditions [7, 8] and is the leading reason for spinal surgery in the elderly [9]. A recent systematic review found the prevalence of symptomatic LSS to be 11% in the general population [10], and almost half of those over the age of 60 experience symptomatic LSS [11]. The number of individuals with disability attributed to LSS is expected to rise globally due to the rapidly increasing population over the age of 60 years [12]. Even with most patients experiencing substantial improvement from surgical intervention, pain and disability persist at long-term follow-up [13]. One possible explanation for the continuing symptoms in these patients may be the presence of comorbid musculoskeletal conditions, as comorbid conditions limiting walking ability, including knee and hip arthrosis, predict worse surgical outcomes [14].

Multimorbidity in the aging population is becoming increasingly recognized as an important health determinant [15–17]. Musculoskeletal pain often occurs in more than one body site [18–20]. An Australian study found 61% of women in the sample had multi-joint pain, with low back pain (35%) and knee pain (27% right, 24% left) as the most common locations, while hip pain was less common (15% right, 16% left) [21]. OA has been shown to be associated with other comorbid conditions [22–24], including other musculoskeletal conditions [24]. In one sample of patients with clinically diagnosed knee OA, 55% reported back pain, and the presence of back pain was associated with worsened levels of pain and disability [25].

LSS can also coexist with other musculoskeletal conditions, including OA [26, 27]. One study found a mean of two comorbidities (less than 20% reported no comorbidities) in LSS patients, with lower limb arthrosis included among the most common comorbidities [28]. Hip-spine syndrome has been described in the literature to define coexisting hip and lumbar spine disorders and was originally developed to describe concomitant degenerative spine and hip disease [29]. Particularly interesting is the relation between hip OA and degenerative LSS, as these conditions share a similar degenerative etiology and radiographic findings of degeneration occur in both the lumbar spine and hip [11, 30–32]. Clinical reports have documented patients with co-occurring LSS and hip OA [27, 33], but the relationship with co-occurring knee OA [34], is relatively unknown. However, there is evidence suggesting knee OA and low back pain commonly co-exist [24] and low back pain has been identified as a risk factor for the development of knee pain in older adults [35]. A recent study also found the presence of radiographic knee OA was associated with all phenotypes of spinal OA (odds ratios ranging from 1.8 to 4.3) [36], which may represent the spinal changes found in LSS.

As the number of individuals with LSS, knee OA, and hip OA rises, it is likely that many older individuals will experience these conditions comorbidly. In fact, the number of individuals living with multimorbidity is increasing as a result of the aging global population [15]. A substantial economic burden has been attributed to multimorbidity in older adults [37] due to functional decline and loss of independence [38]. While it is likely that comorbid LSS and knee and/or hip OA will impact disability levels and healthcare costs, a more developed understanding of the magnitude of this growing health concern is required for informed prioritization and management of these individuals. We are unaware of any formal attempts to estimate the prevalence of multimorbid LSS with knee and/or hip OA. Thus, we do not know how often these conditions co-occur. It is unknown if the relationship between LSS and OA is an incidental imaging finding and unrelated to more severe symptoms and disability or if both conditions have a unique contribution to the health state of patients. As such, multimorbid prevalence estimates using a variety of definitions of LSS and OA are needed.

## Objectives

The overall objective of this systematic review is to estimate the prevalence of multimorbid degenerative LSS with knee and hip OA, respectively.

The primary outcome will be the prevalence of degenerative LSS defined by a combination of clinical evaluation and imaging with co-occurring (i) knee OA and (ii) hip OA.

Secondary outcomes will be the prevalence of degenerative LSS defined by clinical evaluation and co-occurring (i) knee OA and (ii) hip OA and the prevalence of degenerative LSS defined by imaging and co-occurring (i) knee OA and (ii) hip OA.

## Methods

This protocol has been prepared according to the guidelines from the Cochrane Collaboration [39] and reported according to the Preferred Reporting Items for Systematic Reviews and Meta-Analysis Protocols (PRISMA-P) [40], and the populated version is available in Additional file 1. The systematic review protocol is submitted for registration in the international prospective register of systematic reviews (PROPSERO) and is awaiting a registration number.

### Case definitions

All case definitions for degenerative LSS, knee OA, and hip OA will be included in this study. This includes imaging and clinical diagnoses, as well as combinations of imaging and clinical diagnoses. Imaging diagnoses will be based on radiographic, magnetic resonance imaging, or computerized tomography descriptions of narrowing of the central, or lateral canals of the lumbar spine. As no widely accepted gold standard for the clinical diagnosis of LSS exists, this review will include all definitions. Clinical diagnosis will be based on signs and symptoms of LSS, including but not limited to, reduced walking capacity, symptom relief with sitting, and symptom relief with spinal flexion. Moreover, all clinical diagnoses associated with degenerative LSS (neurogenic claudication, radicular type, and mixed types) will be included [41], as they represent the clinical manifestation of central, lateral, and combined central and lateral canal stenosis, respectively.

### Study eligibility criteria

Studies will be included in this systematic review if they meet the following inclusion and exclusion criteria:

#### Inclusion criteria

- Study designs including cross-sectional studies, cohort studies, and randomized controlled trials.
- Studies including adults (18 years or older) with LSS and knee and/or hip OA.
- Studies assessing the prevalence of co-occurring LSS and knee and/or hip OA or presenting sufficient data for estimating the prevalence (number of participants with LSS, number of participants with knee and/or hip OA, and total number of participants).
- Full-text papers published in English in peer-reviewed journals.

#### Exclusion criteria

- Studies including individuals with low back, knee, or hip pain with other origins (e.g., fracture, tumor, inflammatory disease, infection, and lumbar disc herniation).
- Studies including congenital or non-degenerative forms of LSS, without separate data on degenerative LSS.
- Laboratory studies, cadaveric studies, and conference abstracts.

### Search strategy

A comprehensive search for relevant studies was designed in consultation with a health sciences librarian and will be reviewed by a second librarian using the Peer Review of Electronic Search Strategies (PRESS) Checklist [42, 43]. The following bibliographic databases will be searched with no publication date or language limitation, but only articles in English will be included: MEDLINE, EMBASE, CENTRAL, and CINAHL. Forward citation tracking will be performed in Web of Science. Search term groups will be combined covering the following domains: LSS, knee OA, and hip OA. The search terms used for each domain were developed based on previous Cochrane reviews on LSS [44] and knee [45] and hip OA [46]. We included search terms related to low back pain in the LSS search domain to increase the sensitivity of the search strategy. Additional file 2 presents the search strategy designed for MEDLINE. A pilot search has been performed using the search terminology to ensure its all-inclusiveness and that sufficient original articles exist to perform this review. Automated search updates will be set up in each database to ensure the inclusion of the latest publications in the field.

Reference lists of retrieved articles and reviews will be scrutinized. Scientific abstracts presented from 2018 onwards at the International Forum for Back and Neck Pain Research in Primary Care and Osteoarthritis Research Society International World Congress will be reviewed to identify relevant studies. Content experts will be contacted to identify additional studies not captured in the bibliographic database search. Content experts known to members of the study team will be identified and these experts will be asked to identify additional potential content experts. PROSPERO will also be searched for ongoing or recently completed systematic reviews. All studies identified by our search strategy will be retrieved and managed using Endnote X9 (Thomson Reuters, Philadelphia, PA, USA) and Covidence systematic review software (Veritas Health Innovation, Melbourne, Australia).

### Study selection

All retrieved records will be scrutinized in a two-stage screening process by two independent reviewers. Reviewers will first independently screen titles and abstracts according to eligibility criteria, and disagreements will be discussed until consensus or resolved by a third independent reviewer if necessary. Full-text articles of all studies deemed eligible will be retrieved.

In the second stage, the two reviewers will independently screen the full-text articles against the eligibility criteria. Study authors will be contacted for additional information regarding eligibility criteria if necessary. Disagreements will be resolved using consensus meetings or by a third reviewer if consensus cannot be reached. Reasons for excluding full-text studies will be recorded. Absolute agreement and the Kappa coefficient [47] will be calculated for both phases of screening.

### Data extraction

Data from the included studies will be extracted by two authors independently using a standardized data extraction form developed by the authors for this review. Disagreements will be resolved by discussion until consensus or by including a third reviewer. Authors of included studies with missing data will be contacted when additional information is required for extraction. The data extraction form will be tested on ten randomly selected studies from the pilot search and amended accordingly.

Data extraction will include the following:

- First author, publication year, and country.
- Study topic, objectives, and design.
- Time of study, method of data collection, study population, and health care setting.
- Total sample size, participation and response rate, and cohort characteristics (e.g., mean age, age range, sex distribution, ethnicity, and socioeconomic status).
- Case definitions and clinical presentations of LSS.
- Case definitions of knee and/or hip OA.
- Prevalence of LSS and knee and/or hip OA.
- Reports of pain severity and disability levels.
- Information for assessment of methodological quality.

### Risk of bias assessment

Two members of the study team will independently assess the risk of bias of the included studies. Assessment of the risk of bias will be conducted using a modified version of the risk of bias tool for prevalence studies developed by Hoy et al. [48]. Modifications were made to the risk of bias tool for the purpose of this study. All items on the original tool that made specific reference to low back pain were altered to lumbar spinal stenosis and knee and hip osteoarthritis, where applicable. Item 1 was altered from “a close representation of the national population” to “a close representation of the target population” as this review is not concerned with national populations. Item 5 was removed as clinical and imaging information can only be collected directly from participants, and thus irrelevant to the aims of this review. An additional response option “Irrelevant” was added to item 9 for studies that report imaging diagnoses, as imaging findings are not subject to recall limitations. The modifications to items 1, 5, and 9 have been used previously in a prevalence review for LSS [10]. Additionally, item 6 was split into two items, 6a and b, to assess the acceptability of the case definition for (a) lumbar spinal stenosis and (b) knee and hip osteoarthritis. Item 7 was also divided into two items, 7a and b, to assess the reliability and validity of the measurement instrument for (a) lumbar spinal stenosis and (b) knee and hip osteoarthritis. The modified risk of bias tool is presented in Additional file 3.

The modified risk of bias tool will be used for all included study designs (including randomized controlled trials), as only cross-sectional data from these designs will be used in prevalence estimates. Individual items on the risk of bias tool will be rated as “Yes” for low risk of bias or “No” for high risk of bias or if there is insufficient information in the study to allow judgment of the particular item. An overall risk of bias (high, moderate, or low) for each study will be determined based on the consensus agreement of all raters, taking into consideration the responses to each item on this tool.

### Evidence synthesis

**Fig. 1:**
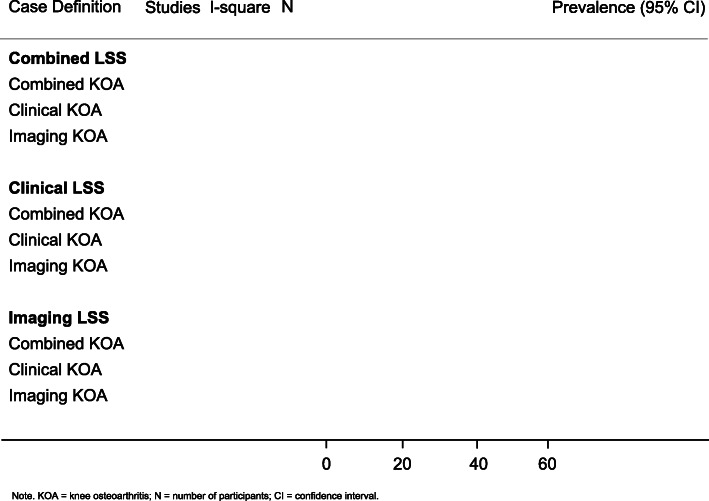
Prevalence estimates of multimorbid degenerative lumbar spinal stenosis and knee osteoarthritis

The selection process will be summarized in a PRISMA flowchart. The results of data extraction and assessment of risk of bias will be summarized in tables. Study and participant characteristics will be reported descriptively. The proportion of participants with LSS reporting co-occurring knee and hip OA will be described as prevalence estimates with 95% confidence intervals. Estimates of the pooled proportions using a random effects model will be calculated, if possible, for the co-occurrence of LSS with knee and hip OA, respectively. Results for prevalence estimates for multimorbid LSS and knee OA will be presented in Fig. 1, while prevalence estimates for multimorbid LSS and hip OA will be presented in a similar figure. If meta-analysis is not possible, a narrative synthesis of included study results will be performed. If it is necessary to perform a narrative synthesis, results will be stratified according to the risk of bias (low, moderate, and high).

Heterogeneity will be evaluated using the *I*^*2*^ statistic [39] and Q index [49]. *I*^*2*^ values of 0% represent no *inconsistency* between the results, and *I*^*2*^ values of 100% represent *maximal inconsistency* between the results in the included studies. The inconsistency can be considered low if *I*^*2*^ is less than 40%, moderate between 30 and 60%, substantial between 50 and 90%, and considerable between 75 and 100% [39]. Publication bias will be investigated using a visual examination of the funnel plot. When there is no publication bias, the funnel plot will have a symmetrical, funnel shape, whereas an asymmetrical funnel plot indicates a publication bias [50].

Meta-regression analyses investigating the impact of LSS clinical presentations (neurogenic claudication, radicular type, and mixed type), sample population (general public, occupational), healthcare setting (hospital, community), and risk of bias (low, moderate, and high risk of bias according to the modified risk of bias tool), as well as the covariates age (mean age in the individual studies) and sex (percentage of female participants in the individual studies), if possible. The impact of self-reported LSS pain severity will be evaluated using the visual analog scale for pain and numeric rating scale for pain (computed individually), if sufficient data is available. Self-reported LSS disability will also be investigated individually using the Swiss Spinal Stenosis Questionnaire, and Oswestry Disability Index, or other disability questionnaire scores, when possible. These potential moderators were chosen for investigation as they have been evaluated in previous and ongoing meta-analyses for LSS [10, 51]. We expect that prevalence is positively associated with increasing age, percentage of female participants, and pain and disability levels. We do not expect prevalence estimates to be impacted by the risk of bias rating of studies or the LSS clinical presentation. A co-variate able to reduce *I*^*2*^ (and thus the between study variance tau-square) will be regarded as important for the prevalence of co-occurrence. All statistical analyses will be performed in Stata 16.1 (StataCorp LLC, College Station, USA).

## Discussion

The discussion will include the strengths and limitations of this review. This review is strengthened by the adherence to recommendations from the Cochrane Handbook [39] and reporting according to the PRISMA-P statement [40]. A detailed search strategy, developed in conjunction with a research librarian, performed in multiple databases also strengthens this review. Additionally, the inclusion of all case definitions of LSS and knee/hip OA will provide a comprehensive review of all available literature and the presentation of separate prevalence estimates by case definition for both LSS and knee/hip OA will increase the clinical value.

This review is not without limitations. The lack of consensus gold standard case definitions for both LSS and OA may limit our ability to draw firm conclusions about the multimorbid prevalence of these conditions. It is likely that clinical diagnoses of LSS and OA are of greater importance than imaging diagnoses due to their relationship with functional impact. However, we have chosen to include all case definitions in this review as we expect there will be limited studies available to estimate the multimorbid prevalence, and we will attempt to provide prevalence estimates for each combination of case definition.

We have chosen to operationalize multimorbidity by simply counting the presence of these conditions, when many other multimorbidity frameworks have been proposed [52] and differing definitions can influence multimorbidity prevalence estimates [53]. It may be that other factors related to multimorbid LSS and OA, such as disability level, health-related quality of life, and others, are of greater importance than the simple presence of these conditions. As this is the first review attempting to estimate the prevalence of multimorbid LSS and OA, the simplest operationalization of multimorbidity was selected. While a more nuanced multimorbidity definition would be ideal, we do not believe sufficient literature exists to perform a review of this nature. This review should be viewed as a starting point aimed at highlighting the potential problem of multimorbid LSS and hip and knee OA, which can be used in the design of more elaborate multimorbidity studies. As such, we have included only English language studies for this review, as we do not expect a large language bias in this research area. However, it is possible relevant studies will not be identified, but it is unlikely the exclusion of a small number of studies will profoundly impact the results of this review.

This review will provide preliminary prevalence estimates of multimorbid LSS and knee and/or hip OA. The results of this review should raise awareness among researchers and clinicians regarding how commonly these conditions coexist and the need to consider multimorbid presentations when assessing and treating patients and evaluating interventions. The generated prevalence estimates should serve as a starting point from which further research can be conducted to better understand the relationship between co-occurring LSS and knee and/or hip OA. The findings will help determine the need for more rigorous epidemiological studies, as well as inform diagnostic and interventional studies for this patient population. It is our hope that the results of this systematic review and meta-analysis will help policymakers better understand the magnitude of this growing healthcare burden, while also helping clinicians and patients access care pathways better suited to manage these complex multimorbid presentations.

## Supplementary information

**Additional file 1.** PRISMA-P Checklist.

**Additional file 2.** MEDLINE Search Strategy.

**Additional file 3.** Risk of Bias Tool.

## Data Availability

The datasets used and/or analyzed during the current study are available from the corresponding author on reasonable request.

## Abbreviations

LSS: Lumbar spinal stenosis
OA: Osteoarthritis
PRESS: Peer Review of Electronic Search Strategies Checklist
PRISMA-P: Preferred Reporting Items for Systematic Reviews and Meta-Analysis Protocols
