# Supplementary material for: Prevalence of multimorbid degenerative lumbar spinal stenosis with knee and/or hip osteoarthritis: protocol for a systematic review and meta-analysis
Source: Syst Rev. 2020 Oct 7;9:232. doi: 10.1186/s13643-020-01478-4 (PMC7542960; doi:10.1186/s13643-020-01478-4)
Supplement: Supplementary file 2 — Additional file 2. MEDLINE Search Strategy. [file 13643_2020_1478_MOESM2_ESM.docx]

**Additional file 2 MEDLINE Search Strategy**

1 exp Spinal Stenosis/

2 spinal stenos*.mp.

3 (lumbar adj5 stenos*).mp.

4 (spin* adj5 stenos*).mp.

5 neurogenic claudication.mp.

6 lumbar radicular pain.mp.

7 exp Cauda Equina/

8 cauda equina.mp.

9 exp Spinal Osteophytosis/

10 spinal osteophytosis.mp.

11 exp Spondylosis/

12 spondylosis.mp.

13 exp Spondylolisthesis/

14 spondylolisthesis.mp.

15 exp Low Back Pain/

16 (low* adj5 back adj5 pain).mp.

17 or/1-16

18 exp Osteoarthritis/

19 osteoarthr*.mp.

20 (degenerative adj2 arthritis).mp.

21 arthros*.mp.

22 or/18-21

23 Knee/

24 exp Knee Joint/

25 knee.mp.

26 or/23-25

27 Hip/

28 exp Hip Joint/

29 hip*.mp.

30 or/27-29

31 (26 or 30) and 22

32 17 and 31
